# Comparison of Potassium-Competitive Acid Blockers and Proton Pump Inhibitors in Patients With Gastroesophageal Reflux Disease: A Systematic Review and Meta-Analysis of Randomized Controlled Trials

**DOI:** 10.7759/cureus.65141

**Published:** 2024-07-22

**Authors:** Demeke E Agago, Najma Hanif, Ariga Sai Ajay Kumar, Muhammad Arsalan, Manpreet Kaur Dhanjal, Lubna Hanif, Calvin R Wei

**Affiliations:** 1 Medicine, Dilchora Hospital, Dire Dawa, ETH; 2 Medicine, Sindh Medical College, Karachi, PAK; 3 Medicine, Bhaskar Medical College, Hyderabad, IND; 4 Internal Medicine, Lady Reading Hospital MTI, Peshawar, PAK; 5 Medicine, Adesh Institute of Medical Sciences and Research, Ludhiana, IND; 6 Medicine, Karachi Medical and Dental College, Karachi, PAK; 7 Research and Development, Shing Huei Group, Taipei, TWN

**Keywords:** systematic review and meta-analysis, efficacy, potassium competitive acid blocker, proton pump inhibitors, gastro-oesophageal reflux disease

## Abstract

This meta-analysis evaluated the efficacy and safety of potassium-competitive acid blockers (PCABs) compared to proton pump inhibitors (PPIs) in treating gastroesophageal reflux disease (GERD). A comprehensive literature search was conducted across multiple databases, and 11 randomized controlled trials comparing PCABs with PPIs were included. The primary outcome was the healing of erosive esophagitis (EE), with secondary outcomes, including relief of heartburn symptoms and adverse events. The analysis included 11 studies and a pooled sample of 4,108 GERD patients. Results showed that PCABs were significantly more effective in healing EE compared to PPIs (OR: 1.67, 95% CI: 1.24-2.24, p<0.01). PCABs also demonstrated a higher rate of complete resolution of heartburn symptoms, although this difference did not reach statistical significance (OR: 1.43, 95% CI: 0.98-2.09, p=0.06). In terms of safety, there was no significant difference in adverse events between PCABs and PPIs (OR: 0.91, 95% CI: 0.79-1.04, p=0.18), including serious adverse events. The superior efficacy of PCABs can be attributed to their unique pharmacological properties, which allow for more rapid and potent acid suppression compared to PPIs. However, the long-term safety profile of PCABs, particularly newer agents, requires further investigation. The study was limited by the predominance of vonoprazan among the PCABs studied and the focus on patients with EE rather than non-erosive reflux disease. In conclusion, this meta-analysis suggests that PCABs are more effective than PPIs in treating GERD, particularly in healing EE, while maintaining a comparable safety profile. Future research should focus on evaluating a wider range of PCABs, assessing their efficacy in non-erosive reflux disease, and investigating their long-term safety in GERD management.

## Introduction and background

Gastroesophageal reflux disease (GERD) is a prevalent gastrointestinal condition caused by the backflow of stomach acid into the oesophagus [1]. According to a recent report, the global prevalence of GERD is 13.3%, with rates of 10.0% in Asia, 15.4% in North America, and 17.1% in Europe [2]. GERD is categorized into erosive esophagitis (EE) or non-erosive reflux disease (NERD), depending on whether esophageal mucosal breaks are detected during an endoscopic examination [3]. The pathophysiology of GERD is complex, involving multiple mechanisms. Lower esophageal sphincter (LES) dysfunction plays a crucial role. The LES is a ring of muscle at the junction of the esophagus and stomach that acts as a barrier to prevent acid reflux [3]. In GERD patients, this sphincter may become weak or relax inappropriately, allowing acid to escape into the esophagus. Transient LES relaxations (TLESRs) are another significant factor [4]. These are brief episodes of LES relaxation that are not related to swallowing. TLESRs can occur spontaneously and are a normal physiological event, but in GERD patients, they occur more frequently and are a major cause of acid reflux [2,3].

GERD significantly impacts patients' quality of life. One major issue is sleep disturbances. The reflux of acid can occur more frequently at night, leading to interrupted sleep and insomnia. This lack of rest can result in daytime fatigue, decreased productivity, and overall poorer physical health [2]. Dietary restrictions also play a crucial role in managing GERD, but they can be burdensome for patients. Many individuals must avoid foods and beverages that trigger symptoms, such as spicy foods, chocolate, caffeine, and alcohol. This can limit their ability to enjoy meals and social gatherings, potentially leading to feelings of frustration and deprivation [2,4]. Untreated or poorly managed GERD can lead to serious complications, including Barrett's esophagus, where the esophageal lining changes and increases cancer risk; esophageal strictures, which cause swallowing difficulties due to narrowed esophagus; and a heightened risk of developing esophageal adenocarcinoma, a potentially fatal cancer [3].

Proton pump inhibitors (PPIs) are the recommended course of treatment for EE according to guidelines [5]. The rate at which EE heals is significantly influenced by both the extent and duration of stomach acid suppression. More effective and prolonged acid suppression reduces the acidity in the esophagus, allowing the damaged esophageal tissue to repair more efficiently and reducing inflammation [6]. PPIs are presently the most potent acid inhibitors that have been licensed for the treatment of EE in both the US and Europe [6]. For patients with severe EE, classified as Los Angeles Classification (LA) Grade C or D, long-term maintenance therapy with PPIs is advised to sustain healing, as nearly 100% of these patients experience recurrence without treatment [6]. However, PPIs have limitations in treating GERD, particularly in addressing atypical and extraesophageal symptoms, as well as typical symptoms [7]. These shortcomings may be attributed to factors such as the variability in PPI metabolism due to cytochrome P450 (CYP) 2C19 polymorphisms and the delayed onset of action caused by the slow absorption of PPIs, which is related to their enteric coating designed to prevent degradation by stomach acid [8].

A potassium-competitive acid blocker (PCAB), a novel class of antisecretory medication that offers more effective suppression of stomach acid than PPIs, is a possible substitute treatment [9]. PCABs inhibit gastric acid secretion by competitively binding to the potassium-binding site of the H+/K+ ATPase enzyme, blocking the final step of acid production in the stomach [10]. This provides a more rapid and potent acid suppression compared to PPI, which requires activation in an acidic environment and can have delayed onset [11]. Advantages of PCABs over PPIs include faster symptom relief, more consistent acid control, and less variability in patient response [12]. Examples of PCABs include vonoprazan, tegoprazan, fexuprazan and revaprazan, which have shown effectiveness in treating conditions such as GERD and peptic ulcers [12].

This meta-analysis aims to rigorously evaluate the efficacy and safety of PCABs in treating patients with GERD. In the present meta-analysis, we included all types of PCABs, including vonoprazan, tegoprazan, and fexuprazan, and assessed the efficacy of these drugs in healing, relieving heartburn symptoms, and safety of drugs compared to PPIs.

## Review

Methodology 

The current review was written in accordance with the 2020 Preferred Reporting Items for Systematic Review and Meta-Analyses (PRISMA) checklist.

Search Strategy 

Comprehensive literature searches were conducted in multiple databases, including PubMed, Embase, and Cochrane Library. The search was conducted by two authors (AK and LH) independently, using keywords and MeSH terms such as "potassium-competitive acid blockers", "PCABs", "proton pump inhibitors", "PPIs", and "GERD" along with their synonyms. The search strategy is attached in the Appendix. The search was conducted from the inception of databases to 15th June 2024. We performed a search irrespective of the publication language. The reference lists of relevant studies were manually screened to identify additional relevant articles. Any disagreement between the two authors regarding study inclusion was resolved through discussion or by consulting a third reviewer (DA) to ensure consensus and accuracy in the selection process.

*Study Selection* 

Studies were selected based on predefined inclusion criteria: randomized controlled trials (RCTs), comparative studies of PCABs and PPIs, included patients with GERD, encompassing NERD and EE. Only RCTs comparing all types of PCABs with PPIs were included. Exclusion criteria included studies without clinical outcomes, observational studies, non-human studies, review articles, case reports, and series. For management, articles were loaded into EndNote X9. To guarantee quality and relevance, two authors (NH, MA) separately first screened the titles and abstracts before conducting full-text reviews. Any differences of opinion among the authors were worked out by conversation or advice from a third reviewer (DA).

Data Extraction and Outcomes 

Data were extracted (MD, LH) using a standardized form developed using Microsoft Excel (Microsoft® Corp., Redmond, WA), capturing information on study design, author name, intervention details, outcomes measured, and results. Two independent reviewers extracted the data to ensure accuracy and resolve discrepancies through discussion. The extracted data were then synthesized to compare the efficacy and safety of PCABs over PPIs.

The primary outcome was the healed EE. Secondary efficacy outcomes included the number of patients with relieved heartburn symptoms. Safety outcomes included adverse events, treatment-related adverse events, and serious adverse events. The risk of bias assessment was assessed using the Cochrane risk of bias assessment tool.

Statistical Analysis 

We performed analysis using RevMan (version 5.4.1; Cochrane Collaboration, London, UK). To compare the effect of PCAB and PPI in patients with GERD, we computed the odds ratio (OR) with a 95% confidence interval (CI). P-values less than 0.05 were considered significant. We used fixed or random-effect models while calculating pooled estimates based on heterogeneity values. Heterogeneity was evaluated using I-square values. In case of significant heterogeneity (I-square>50%), we used a random-effect model. Otherwise, a fixed effect model was used.

Results

**Figure 1:**
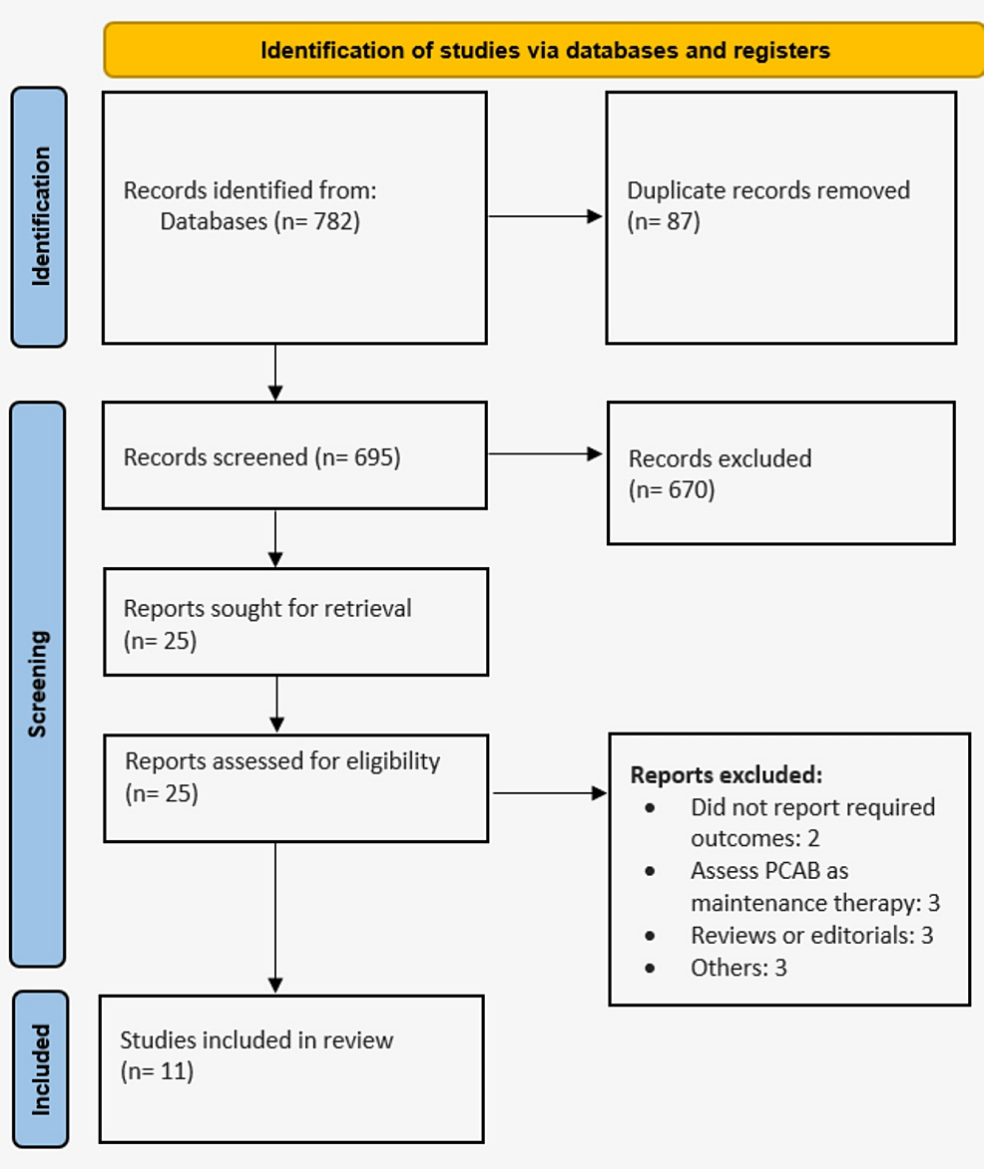
PRISMA flowchart of the study selection

**Figure 2:**
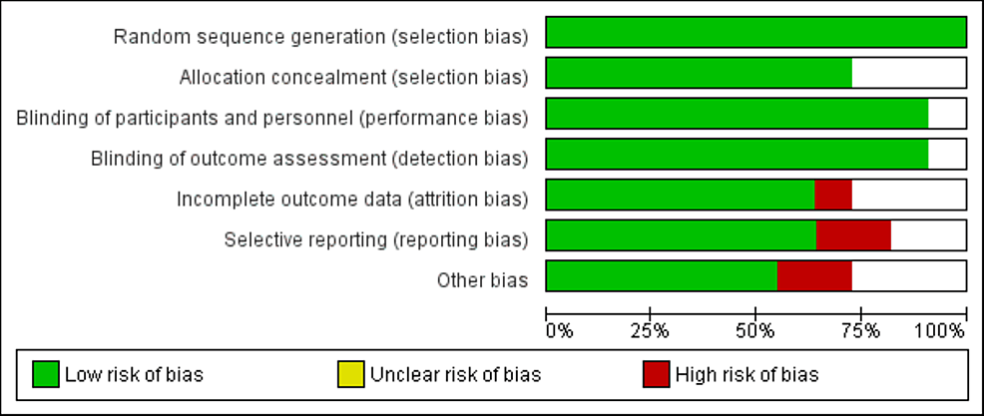
Risk of bias graph

The PRISMA flowchart for research selection is shown in Figure 1. A total of 782 studies were found using the manual hand search and the internet database search. Following the initial screening of 695 records (duplicate records were removed), 25 studies were subjected to full-text screening based on predetermined inclusion and exclusion criteria. Ultimately, this meta-analysis contained eleven papers. The characteristics of the included studies are displayed in Table 1. Vonoprazan was included in seven investigations, tegoprazan was evaluated in three, and fexuprazan was included in one. There were 4,168 GERD patients in the study's pooled sample. Figure 2 presents the quality assessment of the included studies.

**Table 1:** Characteristics of the included studies PCAB: Potassium competitive acid blocker; PPI: Proton pump inhibitors

Author	Year	Groups	Drugs	Sample Size	Dose of PCAB	Follow-up Duration
Ashida et al. [13]	2015	PCAB	Vonoprazan	554	5, 10, 20, 40 mg	8 Weeks
		PPI	Lansoprazole	132		
Ashida et al. [14]	2018	PCAB	Vonoprazan	201	15 mg	8 Weeks
		PPI	Lansoprazole	406		
Ashida et al. [15]	2016	PCAB	Vonoprazan	207	20 mg	8 Weeks
		PPI	Lansoprazole	202		
Cho et al. [16]	2023	PCAB	Tegoprazan	154	25 mg	24 Weeks
		PPI	Lansoprazole	151		
Kim et al. [17]	2023	PCAB	Tegoprazan	22	50 mg	2 Weeks
		PPI	Esomeprazole	24		
Laine et al. [18]	2023	PCAB	Vonoprazan	514	20 mg	8 Weeks
		PPI	Lansoprazole	510		
Lee et al. [19]	2019	PCAB	Tegoprazan	201	50, 100 mg	8 Weeks
		PPI	Esomeprazole	99		
Lee et al. [20]	2022	PCAB	Fexuprazan	107	40 MG	8 Weeks
		PPI	Esomeprazole	111		
Oshima et al. [21]	2018	PCAB	Vonoprazan	16	20 mg	2 Weeks
		PPI	Lansoprazole	16		
Sakurai et al. [22]	2019	PCAB	Vonoprazan	30	20 mg	4 Weeks
		PPI	Esomeprazole	30		
Xiao et al. [23]	2020	PCAB	Vonoprazan	244	20 mg	8 Weeks
		PPI	Lansoprazole	237		

*Rate of Healing of EE* 

**Figure 3:**
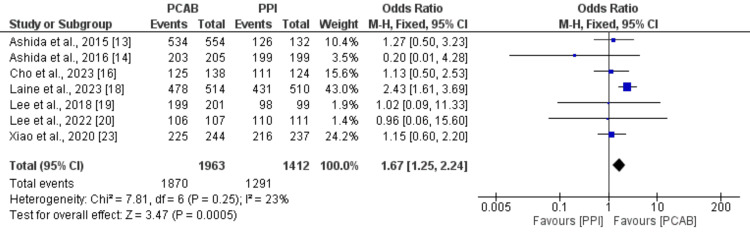
Comparison of the rate of the healing of EE PCAB: Potassium competitive acid blocker; PPI: Proton pump inhibitors Sources: References [13,14,16,18-20,23]

We compared the rate of healing of EE between the two study groups, and the results are shown in Figure 3. The rate of healing of EE in patients who received PCB was higher compared to the patients in the PPI group (OR: 1.67, 95% CI: 1.24-2.24), and the difference between the two groups was statistically significant (p-value<0.01). No significant heterogeneity was reported among the study results (I-square: 23%).

Complete Resolution of Heartburn Symptoms 

Table 2 presents the findings from four of the included studies that examined the total remission of heartburn symptoms between two groups. Patients receiving PCB had a higher rate of completed relief from heartburn symptoms than patients in the PPI group (OR: 1.29, 95% CI: 0.95-1.75), and there was no statistically significant difference between the two groups (p-value<0.06). The study's results showed no discernible heterogeneity (I-square: 32%).

**Table 2:** Comparison of outcomes between the two groups OR: Odds ratio; CI: Confidence interval

Outcome	OR	95% CI	I-square
Resolution of heartburn symptoms	1.29	0.95 to 1.74	32%
Adverse events	0.91	0.79 to 1.04	0%
Serious adverse events	0.66	0.37 to 1.18	0%

Adverse Events and Serious Adverse Events 

We assessed adverse events between two groups by performing a pooled analysis of nine studies [13-20,23]. As shown in Table 2, the rate of adverse events was lower in patients receiving PCB compared to patients in the PPI group, but the difference was statistically insignificant (OR: 0.91, 95% CI: 0.79-1.04, p-value: 0.18). No significant heterogeneity was reported among the study results (I-square: 0%). There was not a significant difference in the two groups' risks of serious adverse reactions (OR: 0.66, 95% CI: 0.37-1.18, p-value: 0.16). The study's results showed no discernible heterogeneity (I-square: 0%).

Discussion

The meta-analysis was conducted to evaluate and compare the effectiveness and safety of PCBs with PPIs. The results of this study indicated that the rate of healing for EE and the alleviation of heartburn symptoms were significantly higher in patients treated with PCB. Nonetheless, in terms of safety, PCB was found to be comparable to PPI. The meta-analysis performed by Cheng et al. indicated that PCB is as effective as PPIs in treating patients with GERD. Subgroup analysis reveals that PCAB surpasses PPIs in effectiveness for patients with severe erosive esophagitis. The safety profiles of vonoprazan and PPIs are comparable [24].

The superior efficacy of PCABs can be linked to their unique pharmacological properties that enable enhanced acid suppression [25]. PCABs offer several advantages, including the stability of the prodrug in acidic environments, a higher affinity for gastric parietal cells, and the ability to remain pharmacologically active even in neutral conditions. On the other hand, PPIs require an acidic environment to become pharmacologically active and are notably less stable in acidic conditions, which can significantly reduce their duration of effectiveness [26].

Among the available PCABs, vonoprazan, revaprazan, and tegoprazan are prominent. Vonoprazan is notable for its rapid onset and sustained acid suppression, making it effective for severe GERD [27,28]. Fexuprazan provides stable acid control and is often used in Asia for peptic ulcer disease [27]. Tegoprazan offers a similar efficacy profile with added benefits in gastric mucosal protection [29]. Safety profiles are comparable, with low incidences of adverse effects, making PCABs a reliable alternative to PPIs [28].

Although PPIs have been used for decades and generally have a favorable safety profile, their long-term utilization has been linked to potential adverse events, including infections, bone fractures, community-acquired pneumonia, chronic kidney disease, vitamin B12 deficiency, and hypomagnesemia [30,31]. The long-term safety of PCABs is less well-known, with the majority of safety data deriving from studies on vonoprazan. Thus far, vonoprazan has demonstrated effective short- to medium-term safety as compared to PPIs. The safety profiles of vonoprazan and lansoprazole were comparable in a phase 3 multicenter clinical trial conducted in Japan; the most frequent adverse event in both treatment groups was nasopharyngitis [15]. Similar safety findings were identified in a meta-analysis by Cheng et al. [32] comparing vonoprazan and PPIs for GERD, with a risk ratio for adverse events of 1.08 (95% CI: 0.96-1.22). Long-term safety data for PCABs are important because GERD is a chronic illness that may last a lifetime. Vonoprazan's safety as a maintenance treatment for patients with healed erosive esophagitis over a five-year period is currently being investigated by the much-awaited "VISION" research [33]. Further research is required to investigate the potential long-term advantages of PCABs.

The benefits of PCABs over PPIs are less clear in NERD compared to EE, as most studies have focused on patients with EE. Trials included NERD patients vary in their eligibility criteria. Some trials, which included all individuals with normal endoscopic findings and typical heartburn symptoms, may have unintentionally included patients with functional heartburn or oesophageal hypersensitivity. More severe suppression of acid would be predicted to have no effect on the latter, but no discernible effect on the former. This might have had an impact on the conclusions and interpretation of a few PCAB trials in cases of suspected "NERD”. Trials that necessitate pathological gastric acid reflux with or without a significant probability of symptom connection prior to enrolment have been shown to more precisely identify patients with real NERD. These trials, however, do not accurately represent standard clinical practice and are costly, time-consuming, and complex to undertake. It is interesting and deserving of more research to examine the possibility of PCABs being used on-demand by NERD patients.

Study Limitations 

The current meta-analysis has certain limitations. Firstly, we were unable to perform a subgroup analysis based on the type of PCAB used, as the majority of studies utilized vonoprazan, with only three studies using tegoprazan and one study using fexuprazan. Secondly, most patients included in the studies had EE, with fewer studies enrolling patients with NERD. Moreover, the studies that included both groups of patients did not assess outcomes separately for each group.

These limitations have important research implications. Future studies should aim to include a more diverse range of PCABs to allow for comprehensive subgroup analyses. Additionally, it is crucial to ensure a balanced representation of both EE and NERD patients and to evaluate outcomes separately for these groups. Addressing these gaps will enhance our understanding of the efficacy and safety of different PCABs across various patient populations.

The findings suggest a potential shift from PPIs to PCABs for treating severe erosive esophagitis due to their superior efficacy. However, long-term safety data for PCABs are essential. The ongoing "VISION" study and further research into the long-term benefits and safety profiles of PCABs will be critical for future clinical guidelines. Additionally, more studies on NERD are necessary to evaluate PCABs' effectiveness in this population and to consider on-demand usage for better patient outcomes.

## Conclusions

This meta-analysis demonstrates that PCABs are more effective than PPIs in healing EE and resolving heartburn symptoms in patients with GERD. PCABs showed a higher rate of healing and symptom relief while maintaining a safety profile comparable to PPIs. The superior efficacy of PCABs can be attributed to their unique pharmacological properties, allowing for enhanced acid suppression. However, long-term safety data for PCABs are still limited, particularly for newer agents. Further research is needed to evaluate the efficacy of PCABs in non-erosive reflux disease and to assess their long-term safety profile in GERD management. It is essential to continue rigorous clinical trials and real-world studies to fully understand their benefits and risks. Healthcare providers should consider individual patient factors when choosing between PCABs and PPIs, keeping in mind the current evidence and the necessity for ongoing monitoring of new therapeutic agents.

## Appendices

Search Strategy for PubMed

(("Vonoprazan"[MeSH Terms] OR "Tegoprazan"[MeSH Terms] OR "Revaprazan"[MeSH Terms] OR "Fexuprazan"[MeSH Terms]) AND ("Proton Pump Inhibitors"[MeSH Terms]) AND ("Gastroesophageal Reflux"[MeSH Terms] OR "Gastroesophageal Reflux Disease"[MeSH Terms] OR "Esophagitis, Peptic"[MeSH Terms] OR "Esophagitis"[MeSH Terms] OR "Esophagitis, Erosive"[MeSH Terms] OR "Non-Erosive Reflux Disease"[MeSH Terms]) OR NERD [MeSH Terms)
